# Functional connectivity abnormalities underlying mood disturbances in male abstinent methamphetamine abusers

**DOI:** 10.1002/hbm.25439

**Published:** 2021-05-03

**Authors:** Ping Jiang, Jiayu Sun, Xiaobo Zhou, Lu Lu, Lei Li, Xiaoqi Huang, Jing Li, Keith Kendrick, Qiyong Gong

**Affiliations:** ^1^ Huaxi MR Research Center (HMRRC), Department of Radiology West China Hospital of Sichuan University Chengdu China; ^2^ Research Unit of Psychoradiology Chinese Academy of Medical Sciences Chengdu China; ^3^ Functional and Molecular Imaging Key Laboratory of Sichuan Province Chengdu China; ^4^ Department of Radiology West China Hospital of Sichuan University Chengdu China; ^5^ Department of Psychosomatics Academy of Medical Sciences & Sichuan Provincial People's Hospital Chengdu Sichuan China; ^6^ Mental Health Center West China Hospital of Sichuan University Chengdu China; ^7^ The Clinical Hospital of Chengdu Brain Science Institute, MOE Key Laboratory for NeuroInformation University of Electronic Science and Technology of China Chengdu China

**Keywords:** anxiety, depressive symptoms, functional connectivity, Methamphetamine, psychoradiology, resting state fMRI

## Abstract

Anxiety and depression are the most common withdrawal symptoms of methamphetamine (METH) abuse, which further exacerbate relapse of METH abuse. To date, no effective pharmacotherapy exists for METH abuse and its withdrawal symptoms. Therefore, understanding the neuromechanism underlying METH abuse and its withdrawal symptoms is essential for developing clinical strategies and improving patient care. The aims of this study were to investigate brain network abnormalities in METH abusers (MAs) and their associations with affective symptoms. Forty‐eight male abstinent MAs and 48 age‐gender matched healthy controls were recruited and underwent resting state functional magnetic resonance imaging (fMRI). The severity of patient anxiety and depressive symptoms were measured by Hamilton anxiety and depression rating scales, which decreased across the duration of abstinence. Independent component analysis was used to investigate the brain network functional connectivity (FC) properties. Compared with healthy controls, MAs demonstrated hypo‐intra‐network FC in the cerebellar network and hyper‐intra‐network FC in the posterior salience network. A whole‐brain regression analysis revealed that FC strength of clusters located in the right rostral anterior cingulate cortex (rACC) within the ventromedial network (VMN) was associated with affective symptoms in the patients. Importantly, the intra‐network FC strength of the rACC in VMN mediated the association between abstinence duration and the severity level of affective symptoms. Our results demonstrate alterations in brain functional networks underlying METH abuse, and that the FC of rACC within VMN serve as a neural substrate in the association between abstinence length and affective symptom severity in the MAs.

## Graphical abstract

In this study, resting state fMRI and independent component analysis methods were used to investigate the whole brain network abnormalities in male methamphetamine abusers and their associations with withdrawal affective symptoms. We found that affective symptoms in the patients were associated with their functional connectivity (FC) strength of clusters located in the right rostral anterior cingulate cortex (rACC) within the ventromedial network, which also served as a mediator in the link between abstinence duration and the severity level of the affective symptoms. Our findings shed light onto alterations in brain functional networks that underlie methamphetamine abuse and its withdrawal psychiatric symptoms and indicate that the rostral ACC may serve as a neural substrate in the association between abstinence length and affective symptom severity in the methamphetamine abusers.

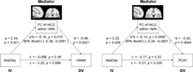

## 1 INTRODUCTION

It is estimated that 35 million people suffer from drug use disorder and need treatment services worldwide (United Nations Office on Drugs and Crime, 2019). The use of methamphetamine (METH), an amphetamine‐type stimulant (ATS), has been increasing in East and South‐East Asia, especially in China. Based on the statistics of the 2018 National Survey on Drug Use in China, METH ranked as the primary drug of abuse in China, accounting for 56.1% of the total number of drug abusers and with ~0.18% nationwide prevalence. Besides the high prevalence of the METH use, the high rates of relapse in METH abusers (MAs) exacerbate the problem resulting in a substantial public health burden worldwide. To date, no available medication‐based interventions can treat this disorder effectively (Gouzoulis‐Mayfrank et al., 2017; London, 2016; United Nations Office on Drugs and Crime, 2019) and therefore understanding the neuromechanism underlying METH abuse is essential for developing clinical strategies and improving patient care.

METH abuse produces a variety of adverse effects, including physiological, affective and cognitive dysfunction, which are usually demonstrated in terms of withdrawal symptoms during abstinence from continuous METH exposure (Hellem, Lundberg, & Renshaw, 2015; Rothman & Baumann, 2003; Thompson et al., 2004). Among the affective symptoms, anxiety and depression are the most common disorders experienced by MAs (Darke, Kaye, McKetin, & Duflou, 2008). Importantly, METH abuse co‐occurring with anxiety or depressive symptoms have greater overall impairment, higher suicide attempts and worse treatment adherence and outcomes relative to those without affective symptoms (Glasner‐Edwards et al., 2008; Glasner‐Edwards et al., 2010). The severity level of the affective symptoms decreases in line with the duration of METH abstinence, but the symptoms can extend from several days to 2–5 years following the last use of METH (Rawson et al., 2002; Zweben et al., 2004). Failure to manage affective withdrawal symptoms also exacerbates relapse of METH abuse (Bao et al., 2013; Zorick et al., 2010). Thus, addressing affective withdrawal symptoms and syndromes in MAs may help to optimize treatment outcomes and develop strategies for relapse prevention.

The affective withdrawal symptoms observed in MAs are presumed to reflect brain functional and structural abnormalities in drug abusers (Rothman, Blough, & Baumann, 2008). Pharmacological and neuroimaging findings suggest that acute METH use activates brain reward systems including limbic, paralimbic, and basal ganglia circuits (including nucleus accumbens [NAcc] and ventral tegmental area), and leads to molecular and neurochemical changes in these brain regions resulting in feelings of pleasure, confidence, and euphoria (Cruickshank & Dyer, 2009; Koob & Volkow, 2016; Panenka et al., 2013). However, repeated use of METH results in neurotoxic effects, such as depletion of monoamine stores, downregulation of monoamine receptors and transporters, and neurite degeneration in the reward system, especially in the striatum and limbic and paralimbic regions, which contribute to the anxiety and depressive symptoms associated with acute withdrawal and protracted abstinence (Cruickshank & Dyer, 2009; Koob & Volkow, 2016; London et al., 2004; Volkow et al., 2001). In addition, chronic drug administration increases stress function in the motivational circuits of the ventral striatum and extended amygdala, and contributes to the negative emotional states in acute withdrawal and protracted abstinence (Koob & Volkow, 2016).

Recently, neuroimaging studies have confirmed that different cortical areas are functionally integrated as brain networks supporting different neurobehavioral functions (Fox et al., 2005), and recent advances in psychoradiology (Gong, Kendrick, & Lu, 2021) including conducting resting‐state functional connectivity (FC) analysis of brain networks to investigate brain functional changes in patients with psychiatric disorders have been widely used (Haber et al., 2020; Lui, Zhou, Sweeney, & Gong, 2016). While previous resting state fMRI (rs‐fMRI) studies mainly focused on dysfunction of a single network or circuit (Ipser et al., 2018; Kohno et al., 2018; Mansoory et al., 2020) and disrupted topological graph properties (Siyah Mansoory, Oghabian, Jafari, & Shahbabaie, 2017) in MAs, whole‐brain network resting‐state FC changes among MAs are still unclear, especially those underlying their anxiety and depressive symptoms.

Our study therefore aimed to use resting state fMRI to characterize abnormal functional brain networks in male MAs compared with age‐gender matched healthy controls (HCs), and also to identify FC changes associated with affective withdrawal symptoms in the MAs. The severity levels of anxiety and depressive symptoms in patients were measured by Hamilton anxiety (HAMA) and depression (HAMD) rating scales. To avoid hypothesis bias, we utilized data‐driven independent component analyses (ICA) to identify whole‐brain networks, and compared the FC differences of each network between the MAs and HCs. In addition, whole‐brain regression analyses were performed to investigate the association between affective symptoms and brain network FC in the patients. Subsequently, mediation analyses were used to explore the potential relationships among affective symptoms, clinical characteristics and brain network FC in the MAs. We hypothesized that (a) FC alterations in brain networks related to reward or stress circuits may underlie the affective symptoms in the MAs, and (b) these network FCs may also mediate the association between the duration of abstinence and affective symptom severity.

## 2 MATERIALS AND METHODS

### 2.1 Participants

This study project was approved by the ethics committee of the West China Hospital of Sichuan University, Sichuan, China for human studies; written informed consent was obtained from each participant. All participants are Han Chinese. Forty‐eight male MAs (mean age, 28.77 years, *SD* 7.66 years) and 48 age‐gender matched HCs (mean age, 28.77 years, *SD* 10.08 years) were enrolled in the study. Patients were consecutively recruited between January and December 2015 and enrolled from Ziyang compulsory isolation and rehabilitation center of Sichuan province, China. Patients participating in our study were required to be at least 16 years old, fulfill the criteria for METH abuse based on the Structured Diagnostic Interview for DSM‐IV Disorders, and be able to understand and complete the measurements. Exclusion criteria included: (a) any psychoactive substance dependence or abuse other than METH or nicotine, or a history of mental disorders before METH abuse; (b) current severe medical diseases requiring in‐patient treatment or frequent medical visits; (c) current use of medications that affect the hemodynamic response such as insulin and thyroid medication; (d) history of head injuries with loss of consciousness and other neurological disorders, such as Parkinson's disease and stroke; (e) any contraindications to MRI measurement (including claustrophobia, cardiac pacemaker or metal implants, etc.).

Age‐gender matched HCs were recruited from posters and flyers distributed at West China Hospital of Sichuan University and from the imaging database of Huaxi Magnetic Resonance Research Center. HC participants were excluded if they had any drug use history other than nicotine or neurologic or psychiatric illness as assessed by medical records or structural brain defects on T1‐weighted images.

### 2.2 Drug use and affective symptom measures

A detailed interview including questions on socio‐demographics, drug use history, and anxiety and depressive symptoms were recorded by experienced psychiatrists. The HAMA and HAMD clinical rating scales were performed by the interviewer to assess the anxiety and depressive symptoms of the MAs. The HAMA is a 14‐item questionnaire which includes both psychic and somatic anxiety items with the total score ranging from 0 to 56 (Hamilton, 1959; Vaccarino, Evans, Sills, & Kalali, 2008). The HAMD is a 17‐item questionnaire with the total score ranging from 0 to 50 (Hamilton, 1960). For both HAMA and HAMD scales higher total score indicate more severe anxiety or depressive symptoms.

### 2.3 MR imaging acquisition

All participants underwent a rs‐fMRI examination by using a 3.0 T system (Tim Trio; Siemens Healthineers, Erlangen, Germany) equipped with a 12‐channel phased‐array head coil. A gradient‐echo echo‐planar imaging sequence was used for obtaining blood oxygen level–dependent–sensitive MR images with the following parameters: repetition time (TR) = 2000 ms; echo time (TE) = 30 ms; flip angle = 90°; slice thickness = 5 mm; intersection gaps, none; matrix size = 64 × 64; field of view = 240 × 240 mm^2^; voxel size = 3.75 × 3.75 × 5 mm^3^. Each brain volume comprised 30 axial sections, and each functional imaging session contained 205 image volumes, resulting in a total imaging time of 410 s. Participants were instructed to keep their eyes closed and to not focus their thoughts on anything in particular during the acquisition. Earplugs were used to eliminate scanning noise and foam pads to minimize participant head motion. A three‐dimensional T1‐weighted image was acquired by using a spoiled gradient‐recalled echo sequence with the following parameters: TR = 1900 ms; TE = 2.26 ms; flip angle = 9°; matrix size = 256 × 256; field of view = 256 × 256 mm^2^; slice thickness = 1 mm; number of axial sections = 176 slices; voxel size = 1 × 1 × 1 mm^3^. An experienced neuroradiologist (J. S.) verified the structural image quality and evaluated scans for clinical abnormalities.

### 2.4 Resting state fMRI image preprocessing

Preprocessing of individual rs‐fMRI data with the first five volumes excluded consisted of brain extraction, motion correction, slice timing correction, high‐pass temporal filtering equivalent to 100 s (0.01 Hz) and spatial smoothing (5 mm FWHM Gaussian kernel). The global signal was not regressed out. Functional MRI data were registered to the individual's structural image and the 2 mm MNI152 standard space template using Boundary‐Based Registration (BBR) method implemented in FMRIB's Linear Image Registration Tool (FLIRT). The FMRIB's ICA‐based Xnoiseifier—FIX (v1.061 beta) (Griffanti et al., 2014; Salimi‐Khorshidi et al., 2014) was applied on each individual's rs‐fMRI data to largely control for any influences of head motion and other nuisance noise (e.g., respiration, cardiac pulse), and to produce cleaned data sets for the subsequent analyses. The level of head motion related noise was significantly reduced (comparison between the mean framewise displacement [FD] before and after denoising in each group both *p* < .0001), but without significant group differences either before or after denoising (both *ps* > .05). Full details of the cleaning steps are provided in the Supplemental Materials.

To create the spatial templates, the cleaned individual resting state data of the 48 HCs were fed into the MELODIC package in FSL (https://fsl.fmrib.ox.ac.uk/fsl/fslwiki/MELODIC) for group‐level decomposition by a temporal concatenation approach with a determined number of output components (i.e., 25 components) (Beckmann & Smith, 2004). After visual inspection and comparison with other templates (e.g., templates in Shirer, Ryali, Rykhlevskaia, Menon, & Greicius, 2012 and Smith et al., 2009) (Shirer et al., 2012; Smith et al., 2009) using spatial correlation analysis by fslcc, 19 components were identified as brain networks, including visual, auditory, sensorimotor, default mode, fronto‐parietal, dorsal attentional and salience networks, and so forth. (See Figure S1). The naming of the networks in the current study was based on previous reports (Huang et al., 2015; Jiang et al., 2018) and on the correlation analysis with other reported brain networks in Shirer et al., 2012 and Smith et al., 2009. The obtained brain networks were used as spatial templates for subsequent analyses.

### 2.5 Group comparisons of brain network functional connectivity

The between group analysis of the rs‐fMRI data were conducted by dual regression and permutation tests (Nickerson, Smith, Ongur, & Beckmann, 2017; Winkler, Ridgway, Webster, Smith, & Nichols, 2014) that allow voxel‐wise comparisons of FC patterns. The spatial templates were used to generate subject‐specific versions of the spatial maps and associated time‐series, using dual regression. First, for each subject, the spatial templates were used as spatial regressors in a multiple regression analysis against the cleaned individual 4D data set. This results in a set of subject‐specific timeseries, one per spatial template. Next, those timeseries were used as temporal regressors, again in a multiple regression analysis against the same 4D data set, resulting in a set of subject‐specific spatial maps, one per spatial template. Finally, FSL's Randomize nonparametric permutation‐testing tool (10,000 permutations) was used to test for statistically significant group differences of intra‐network connectivity controlling for demographic variables, that is, age and education years. A threshold‐free cluster enhancement (TFCE) method was used for thresholding and family‐wise error (FWE) correction with alpha level of .05 for controlling multiple comparisons across voxels of the whole brain (Smith & Nichols, 2009). Individual mean z‐scores derived by dual regression representing within‐network FC strength were extracted for subsequent statistical analysis.

Between‐network FC was examined with the FSLNets toolbox (http://fsl.fmrib.ox.ac.uk/fsl/fslwiki/FSLNets) which uses the subject‐specific timeseries of each spatial template from the dual regression analysis to generate a 19 × 19 matrix of between‐network connection strengths for each subject by L1 regularized partial correlation. The group comparisons of between‐network connectivity strengths were carried out for rs‐fMRI data by permutation tests with FWE multiple comparison correction (thresholded at *p* < .05) (Smith et al., 2013) and with age and education years as covariates. The transformed z‐scores for each subject calculated by the L1 regularized partial correlation analyses in FSLnets representing between‐network FC strength were used for subsequent statistical analysis.

A detailed pipeline for the group comparison of FC between the MAs and HCs is provided in the Supplemental Materials.

### 2.6 Relationship between affective symptoms and FC in the MA group

In the MA group, a group ICA was performed in MELODIC. Dual regression with the same spatial templates described above was used to generate subject‐specific versions of the spatial maps and associated time‐series. Subsequently, a General Linear Model (GLM) with randomized analysis (10,000 permutations) was applied to estimate correlations between affective symptoms (i.e., HAMA and HAMD scores) with (a) the voxel‐wise intra‐network FC strength, and (b) between‐network FC strength using FWE with alpha level of .05 to correct for multiple comparisons.

Individual mean z‐scores representing intra‐network FC strength of the clusters and between‐network FC strength associated with affective symptoms were calculated for subsequent statistical analysis.

### 2.7 Statistical analysis

Correlation analyses in SPSS software (https://www.ibm.com/analytics/spss-statistics-software) were used to investigate the potential relationships between the affective symptoms (i.e., HAMA and HAMD scores) and (a) clinical measurements, (b) intra‐network and between‐network FC strength (z‐scores) demonstrated group differences between the MAs and HCs, iii) FC strength associated with HAMA and HAMD scores. Statistical significance for correlation analyses was set a threshold at *p* < .05 without multiple comparison correction.

### 2.8 Mediation analysis between FC and affective symptoms measures

To examine the indirect effect of resting‐state brain network FC strength on the association between clinical measurements of METH use, duration of abstinence and anxiety or depression scores, we conducted mediation analyses using the SPSS PROCESS v3.4 with a 5,000 bias‐correction bootstrapping approach (Hayes & Preacher, 2014; Preacher & Hayes, 2008). Mediation analysis is a path analysis used to statistically evaluate how the independent variables (IVs) transmit their effects on the dependent variables (DVs) through potential intervening variables or mediators (M) (Hayes, 2018). To this end, the clinical measurements and duration of abstinence were considered as the IVs, HAMA/HAMD scores‐associated intra‐ or between‐network FC z‐scores were considered as the M and the anxiety or depression levels (HAMA/HAMD scores) were considered as the DVs. Age and education years were used as the covariates in these mediation analyses.

### 2.9 Exploration analysis for the affective symptoms

Since there is strong association between HAMA and HAMD scores, we used the dimension reduction method—principle component analysis (PCA)—to extract the first component (PCA1) for the two scores to represent the affective symptoms of the patients. Thereafter, a whole brain regression analysis was conducted to estimate the PCA1 value‐related FC of brain networks. A subsequent mediation analysis was used to evaluate the relationship between the clinical measurements, PCA1 values and the related brain network FC strength after controlling for age and education years. Statistical significance was set at threshold of *p* < .05.

## 3 RESULTS

### 3.1 Demographics, drug use, and affective measurements

Table 1 lists the descriptive statistics for sample characteristics in this study. The MA and HC groups were similar in age (*p* > .10), but differed significantly in education years (*p* < 0.001). All MAs had ceased using METH for an average of 114.56 days (SD = 116.20 days) before taking part in the study.

**TABLE 1:** Demographic information of the MA and HC groups

	MA (*N* = 48)	HC (*N* = 48)
***Demographics***		
Age (years) (± *SD*)	28.77 ± 7.66	28.77 ± 10.08
Education (years) (± *SD*)	8.27 ± 3.68	11.96 ± 2.96^***^
***Affective symptom severity***		
HAMA scores (± *SD*)	3.56 ± 5.09	–
HAMD scores (± *SD*)	4.90 ± 5.25	–
***METH use***		
Abstinence (days) (± *SD*) (range)	114.56 ± 116.20 (12–400)	–
Use duration (months) (± *SD*)^a^	47.74 ± 39.62	–
Mean dose (g/time) (± *SD*)	0.39 ± 0.30	–
Total amount (kg) (± *SD*)^a^	1.05 ± 2.10	–
Age of first use (years) (± *SD*)^a^	24.66 ± 8.20	–

Abbreviation: g, gram; HAMA, Hamilton anxiety rating scale; HAMD, Hamilton depression rating scale; HC, healthy control; kg, kilogram; MA: methamphetamine abuser; METH: methamphetamine; *SD*, standard deviation.

^a^ *N* = 47.

^***^ *p* < .001.

The MAs had a mean HAMA score of 3.56 and a mean HAMD score of 4.90. The correlation analysis showed that the HAMA scores were significantly correlated with the HAMD scores (Spearman *r* = .82, *p* < .0001), and both of them were significantly correlated with the number of days of abstinence of MAs (HAMD, Spearman *r* = −.38, *p* = .0074; HAMA, Spearman *r* = −.31, *p* = .0034) (Figure S3).

The dimensional reduction analysis conducted by PCA for affective symptoms showed that the first dimension of PCA (PCA1) accounted for 91.14% of the total variance of the anxiety and depression scores. The correlation analysis showed that the PCA1 value was significantly correlated with HAMA scores (Spearman *r* = .98, *p* < .0001), HAMD scores (Spearman *r* = .89, *p* < .0001) as well as the number of days of abstinence (Spearman *r* = −.36, *p* = .012) in the MAs.

### 3.2 Group differences in brain‐network functional connectivity

**FIGURE 1:**
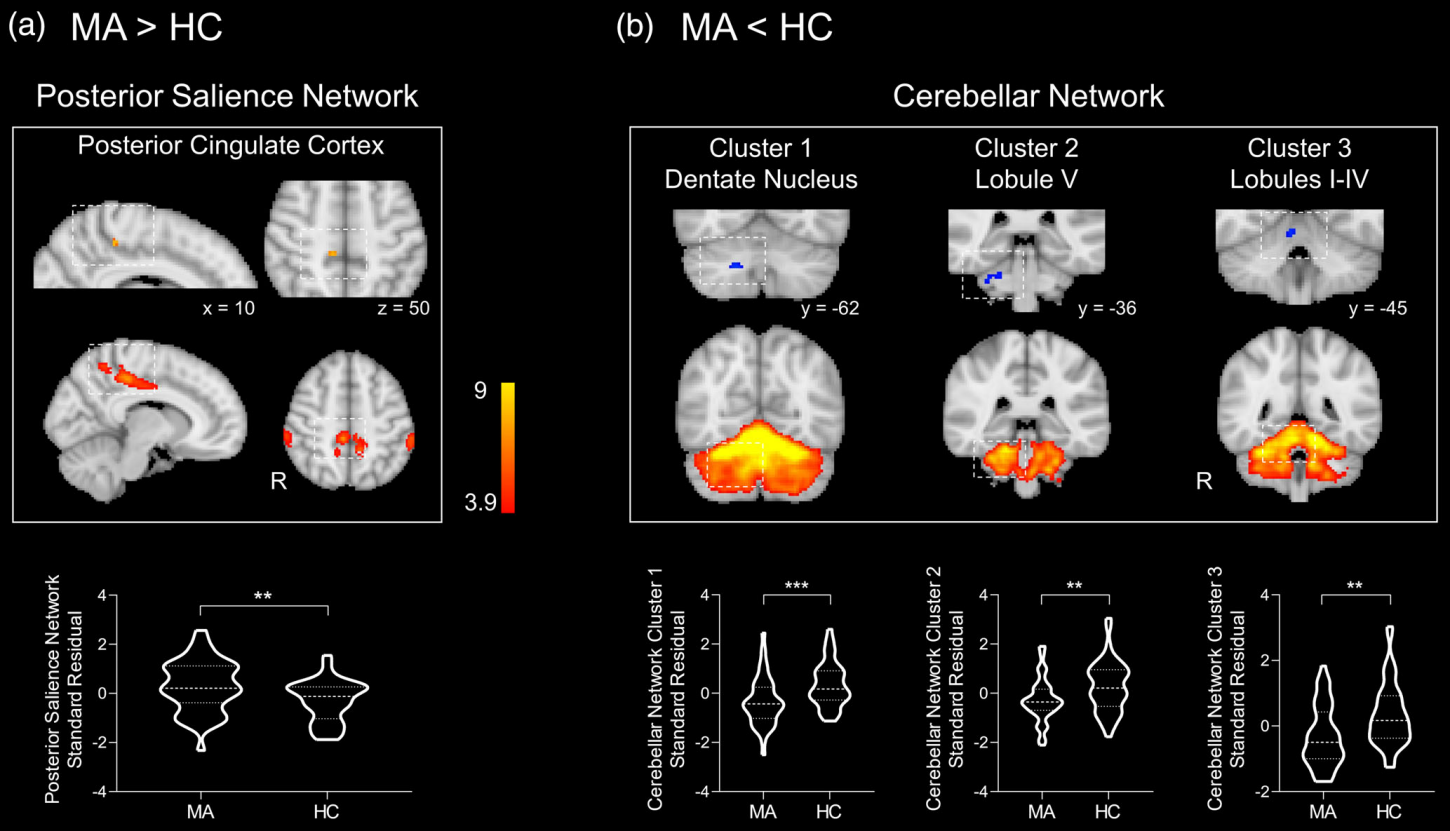
Group differences in brain network FC using age and education years as covariates. Compared with HC, the MA group demonstrated a significantly higher intra‐network FC for the posterior cingulate cortex in the posterior salience network (a) and a lower intra‐network FC for the cerebellum in the cerebellar network (b). The graphs illustrate the standard residual value of intra‐network FC (z scores) after regressing out age and education years as covariates. FC, functional connectivity; HC, healthy control; MA, methamphetamine abuser; R, right. **p < .01; ***p < .001

We defined 19 brain networks from the rs‐fMRI data of the HCs (Figure S1) which were used as spatial templates. Age and education years were used as covariates in the group comparison analyses. Results showed that the MAs relative to HCs had a significantly higher intra‐network FC for the posterior cingulate cortex (PCC) in the posterior salience network and a lower intra‐network FC for the cerebellum in the cerebellar network (*p* < .05, FWE corrected, cluster size >10 contiguous voxels) (Figure 1). Table 2 lists the brain areas and peak voxel coordinates within the networks that showed significant group FC differences. No significant group differences were found in between‐network FC analysis (*p* > .05). No significant correlations were found between FC demonstrated group differences and the severity levels of affective symptoms in the MAs (*p* > .05).

**TABLE 2:** Clusters demonstrate significant group differences and association with affective symptoms in intra‐network FC in the MAs

*Group differences in intra‐network FC with age and education years as covariates*					
		Peak voxel			
Brain networks	Regions	Vox	x	y	z
*MA > HC*					
Posterior salience network	**Right precuneus/PCC**	16	10	−34	50
*HC > MA*					
Cerebellar network					
Cluster 1	**Right dentate nucleus**	152	18	−62	−38
Cluster 2	**Right lobule V**: Lobules I‐IV	51	30	−36	−32
Cluster 3	**Right lobules I‐IV**	42	6	−45	−18

*Significant negative association between intra‐network FC in VMN and affective symptoms in the MAs*					
		Peak voxel			
Affective symptoms	Regions	Vox	x	y	z
*HAMA*	**Right rostral ACC**	13	12	38	4
*HAMD*	**Right WM**: FP, ParaCG, rostral ACC, medPFC	81	16	54	−8
*PCA1*					
Cluster 1	**Right FP**: medPFC, ParaCG	140	16	54	−8
Cluster 2	**Right WM**: ACC, left/right subcallosal cortex, left ParaCG	154	8	28	−6
Cluster 3	**Right WM**	67	20	24	−8
Cluster 4	**Left medPFC**	17	−6	42	−16
Cluster 5	**Right rostral ACC**: WM	24	12	38	4

*Note*: The MNI coordinates of the peak voxel and number of voxels in the cluster are reported to express group differences in intra‐network FC and significant associations between intra‐network FC and affective symptoms in the MAs. The brain area corresponding to peak voxel is in bold and the anatomical areas included in the cluster are written in regular font. The clusters were FWE corrected and thresholded at *p* < .05 with larger than 10 contiguous voxels.

Abbreviations: ACC, anterior cingulate gyrus; FC, functional connectivity; FP, frontal pole; HAMA, Hamilton Anxiety Rating Scale; HAMD, Hamilton Depression Rating Scale; MA, methamphetamine abuser; medPFC, medial prefrontal cortex; ParaCG, paracingulate gyrus; PCC, posterior cingulate cortex; VMN, ventromedial network; Vox, number of voxels; WM, white matter.

### 3.3 Associations between affective symptoms and brain‐network FC in the MA group

**FIGURE 2:**
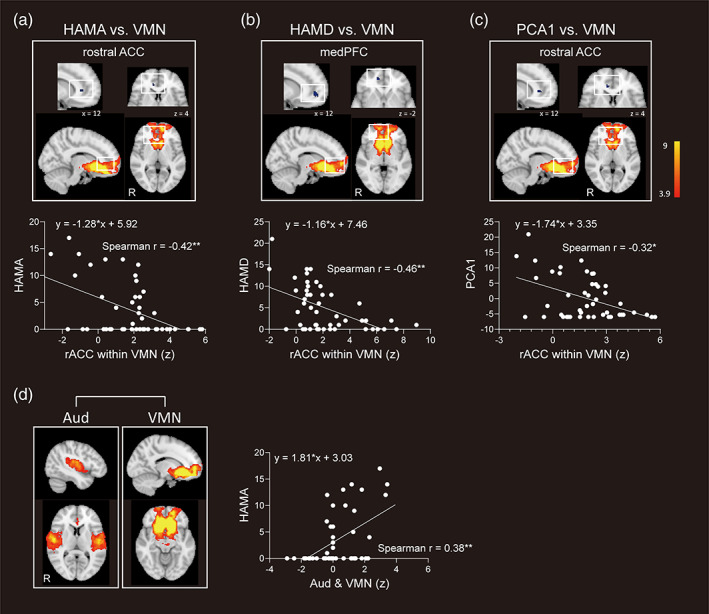
Intra‐network and between‐network FC showing significant correlations with affective symptoms in the MAs. The figures and graphs demonstrate significant correlations between (a) HAMA, (b) HAMD scores and the intra‐network FC of clusters within VMN; (c) one of the five clusters in the VMN showing significant correlation with PCA1 value; (d) significant correlation between HAMA scores and coupling between auditory and VMN networks. Aud, auditory network; medPFC, medial prefrontal cortex; rACC, rostral anterior cingulate cortex; VMN, ventromedial network; HAMA, Hamilton Anxiety Rating Scale; HAMD, Hamilton Depression Rating Scale; PCA1, the first component of principle component analysis; R, right. *p < .05; **p < .01

Whole‐brain regression analyses were conducted to detect potential relationships between brain‐network FC and affective symptoms as estimated by the HAMA, HAMD, and PCA1 scores in the MA group. Significant negative correlations were revealed between HAMA, HAMD scores and intra‐network FC in the ventromedial network (VMN) for clusters in the right rostral ACC (including part of BA 24 and 25) and medial prefrontal cortex (medPFC) (including part of BA 24, 25, and 32) respectively (*p* < .05, FWE corrected, see Table 2 and Figure 2).

Correlation analysis showed that higher intra‐network FC of the right rACC in the VMN was associated with lower HAMA (Spearman *r* = −.42, *p* = .0032) and HAMD scores (Spearman *r* = −.46, *p* = .0010) in the MAs (Figure 2).

Using the PCA1 to represent affective symptoms, the whole‐brain regression analysis found five clusters in the VMN exhibiting a significant negative correlation with the PCA1 value in the MAs (*p* < .05, FWE corrected, see Table 2 and Figure S2). The association between the FC of the cluster located in rACC within the VMN and PCA1 for the MA group (Spearman *r* = −.32, *p* = .025) is illustrated in Figure 2.

For between‐network FC, coupling between the auditory network and VMN demonstrated a significant positive correlation with HAMA scores in the MAs (Spearman *r* = .38, *p* = .0071) (Figure 2).

### 3.4 Intra‐network FC of rACC in VMN linking abstinent days and affective symptoms

**FIGURE 3:**
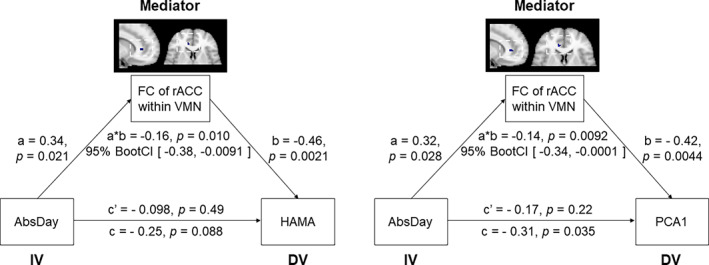
The FC for rACC within the VMN significantly mediated the association between abstinence duration and affective symptoms after controlling for age and years of education in the MAs. The FC of right rACC in the VMN (mediator) mediates the association between abstinence duration (IV) and HAMA scores (DV) and PCA1 value (DV). rACC, rostral anterior cingulate cortex; VMN, ventromedial network; AbsDay, abstinence days; FC, functional connectivity; HAMA, Hamilton Anxiety Rating Scale; METH, methamphetamine; PCA1, the first component of principle component analysis; DV, dependent variable; IV, independent variable

The correlations between number of days of abstinence and HAMA and HAMD scores were significant in the MAs (Figure S3). Mediation analyses were performed to investigate whether FC strengths of the brain networks underlie the association between affective symptoms and the duration of abstinence after controlling for age and years of education. The results revealed that the association between affective symptoms and the intra‐network FC of right rACC in VMN significantly mediated the effect of abstinence duration on HAMA scores (*β* = −.16, *p* = .010) and the PCA1 value (*β* = −.14, *p* = .0092) (Figure 3). The detailed results of the mediation analyses are illustrated in Table S1 and S2.

## 4 DISCUSSION

In this study, we investigated differences in the resting state FC of whole‐brain networks between male MAs and age‐gender matched HCs. The MAs compared with HCs demonstrated hyper‐intra‐network connectivity in the posterior salience network and hypo‐intra‐network connectivity in the cerebellar network. Furthermore, the intra‐network FC strength of different clusters located in the right rACC within the VMN was associated with anxiety and depressive symptoms in the MAs. Importantly, the intra‐network FC of the rostral ACC in the VMN mediated the association between abstinence duration and the level of affective symptoms. Overall, these findings suggest that the FC of brain networks is disturbed in the male MAs during abstinence and that the FC of rostral ACC within the VMN serves as a neural substrate underlying the link between the abstinence length and the affective symptoms in the male abstinent MAs.

### 4.1 Brain network FC differences between MAs and HCs

Our findings revealed abnormalities in intra‐network FC in the PCC of posterior salience network and the cerebellum of cerebellar network in the MAs compared with HCs. The finding of significant hyper‐intra‐network FC of the right PCC in posterior salience network in the MAs compared with HCs was in line with previous studies reporting structural deficits and metabolic increase in this region in chronic MAs (London et al., 2004; Thompson et al., 2004). The posterior salience network, including bilateral supramarginal gyrus, parietal operculum cortex, precuneus and PCC extending to the middle portion of cingulate and insular cortices, is responsible for processing behaviorally salient events and for task initiation and switching between introspection and executive function (Dunlop, Hanlon, & Downar, 2017; Sridharan, Levitin, & Menon, 2008). The PCC is anchored within the posterior salience network and proposed as a functional integrative hub playing an important role in cognitive function by mediating information flow around the brain (Leech & Sharp, 2014). Based on the PCC's predominant contribution to cognitive function, we postulate that the hyper‐intra‐network FC in the PCC reported here may underlie a variety of cognitive deficits in MAs (Darke et al., 2008). In line with this implication, a task fMRI study has provided evidence that the PCC exhibits higher activation in the context of the Stroop effect in MAs compared with the controls (Ghavidel et al., 2020).

Interestingly, we also found three clusters located in the right dentate nucleus, cerebellar lobules I–IV and V in the cerebellar network demonstrating hypo‐intra‐network connectivity in the MAs compared with HCs. The primary function of cerebellum has been recognized as being involved in motor function and anatomical tract tracing in nonhuman primates (Kelly & Strick, 2003) together with functional neuroimaging observations in humans (Buckner, Krienen, Castellanos, Diaz, & Yeo, 2011) have revealed that the anterior lobe of the cerebellum (including lobules I‐IV and V) is anatomically and functionally mapped to the motor cortex. Clinical observations also showed that right cerebellar lobules I–IV and V demonstrated reduced FC with motor cortex in patients with essential tremor compared with healthy controls (Buijink et al., 2015). Therefore, the hypo‐intra‐network connectivity we observed in the lobules I‐IV and V of cerebellum may imply METH‐induced motor deficits in the MAs including stereotypic motor activity and locomotor hyperactivity (Ferrucci et al., 2007; Seiden, Sabol, & Ricaurte, 1993). On the other hand, recent accumulating evidence has revealed that the cerebellum is also engaged in other high‐level functions, such as emotional memory and experience (Sacchetti, Baldi, Lorenzini, & Bucherelli, 2002; Schutter & van Honk, 2006) and cognitive functions (Kuper et al., 2011). Studies on nonhuman primates have demonstrated that the cerebellar dentate nuclei structurally project to, and are functionally related to, the prefrontal cortex involved in working memory (Middleton & Strick, 2000), while animal model (Bauer, Kerr, & Swain, 2011) and patient studies (Thoma, Bellebaum, Koch, Schwarz, & Daum, 2008) suggest that lesions in cerebellar dentate nuclei result in motivational deficits. Our finding of hypo‐intra‐network connectivity in the cerebellar dentate nuclei may therefore reflect cognitive deficits usually seen in MAs, such as in executive function and working memory (Potvin et al., 2018). Moreover, our findings are also in line with observations suggesting the involvement of the cerebellum in many of the brain functions affected in drug addiction (Miquel et al., 2016; Moulton, Elman, Becerra, Goldstein, & Borsook, 2014). In addition, neuroimaging studies have found that the cerebellum responds robustly to acute drug exposure and craving for the psychostimulants cocaine and methylphenidate (Moulton et al., 2014). In cocaine abusers, cue‐elicited craving is also correlated with increases in the regional metabolic rate for glucose in the cerebellum (Grant et al., 1996). Together with our finding of hypo‐intra‐network connectivity of cerebellum in the MAs these observations indicate an important detrimental influence of METH on cerebellar function in humans.

### 4.2 Affective symptom association with brain network FC in MAs

In line with previous reports (Bao et al., 2013; Zorick et al., 2010; Zweben et al., 2004), we found anxiety and depressive symptoms co‐occurred frequently in the MAs, and some individuals continued to experience these affective symptoms from weeks to months after abstaining from METH use. Our correlation analysis also revealed that the severity of these affective symptoms decreased with increasing durations of METH abstinence.

To further explore affective symptom‐related brain networks, we conducted whole‐brain regression analyses using the severity level of affective symptoms as regressors in the MA group. Results showed that the intra‐network FC strengths of the clusters located in the rostral ACC (including partial of perigenual and subgenual ACC) and medial PFC within the VMN were negatively associated with the severity of anxiety and depressive symptoms, as well as the first principal component of the affective symptoms (PCA1).

The VMN includes the temporal pole, medial orbitofrontal cortex, ventromedial prefrontal cortex, rostral ACC, frontal pole and subcortical regions including the NAcc and striatal cortex, which correspond to the classic reward pathway and function in mediating reward, value assignment and incentive salience (Dunlop et al., 2017). Our finding supported our hypothesis and previous reports that repeated METH abuse results in dysfunction in the reward system, especially in limbic regions, which contribute to affective symptoms in abstinent patients (Koob & Volkow, 2016). Addictive patients usually demonstrate distorted incentive salience (Goldstein & Volkow, 2011), which is also a common feature shown in patients with depression (Dunlop et al., 2017). For example, substance use disorder patients attribute excessive salience to drug and drug cues and decreased sensitivity to nondrug reinforcers, which links to abnormal activation of the VMN (Diekhof, Falkai, & Gruber, 2008; Goldstein & Volkow, 2011). Similarly, the VMN of depressive patients responds to negative rather than positive affective stimuli indicating that these patients have acquired aberrant incentive salience. The shared neural substrates of inappropriate VMN function that result in pathological incentives in addictive and depressive patients may account for the high comorbidity prevalence of these two disorders and the tendency for symptoms of one disorder to exacerbate those of the other. On the other hand, abstinent MAs demonstrate decision‐making dysfunctions with hypersensitivity to immediate gains rather than long‐term outcomes, which is also consistent with dysfunction in the brain reward system (Paulus et al., 2002). Similarly, abnormal decision‐making links to the presence of prominent depressive symptoms in mood disorders, which ascribes to a reward processing impairment, characterized by a hyposensitivity to positive outcomes in depression (Mukherjee, Filipowicz, Vo, Satterthwaite, & Kable, 2020; Whitton, Merchant, & Lewandowski, 2020) and anxiety disorders (Reilly et al., 2020).

In terms of a key specific brain region demonstrating abnormal FC in the VMN, the rostral ACC—an important node in the VMN—receives a dense dopaminergic innervation (London et al., 2004) which has been implicated in emotional processes, and dysfunction of this region occurs in major depressive disorders (Drevets, 2000). Previous PET studies have demonstrated that negative affective states, including anxiety and depressive symptoms, are associated with abnormal regional metabolism in the rostral ACC (London et al., 2004), which could serve as an index for predicting eventual medication treatment response in major depression (Mayberg, 2002). In addition, our finding that the rostral ACC exhibited functional associations with affective symptoms in the MAs has also been reported in treatment‐resistant depression patients (La Torre et al., 2020). This could possibly explain why no pharmacological approaches to date have been found to be effective in treating anxiety (Hellem, 2016) or depressive (Hellem et al., 2015) symptoms in MAs, while antidepressant medications can help in reducing METH use (Siefried, Acheson, Lintzeris, & Ezard, 2020). Importantly, we also observed that the intra‐network FC of rostral ACC in the VMN served as a mediator in the link between abstinence duration and the severity of anxiety symptoms, as well as the level of the principle component representing anxiety and depressive symptoms. These findings indicate that increased intra‐network FC of rostral ACC in VMN may underlie the reduction of affective symptoms as a function of abstinence duration. In addition, based on the dense dopaminergic innervation of rostral ACC, the increased intra‐network FC may indicate the physiological recovery of damaged dopaminergic nerve terminals and dopamine transporters with prolonged abstinence in MAs (Rusyniak, 2013). The mediation results further indicate that individual differences in the FC of rostral ACC mediate abstinence duration associated improvements in affective symptoms.

Interestingly, we noted that the brain regions demonstrating FC differences between groups and associated with affective symptoms were located in the right but not the left hemisphere of the brain, which is in line with a previous study reporting right lateralization of reinforcing and conditioned drug responses in cocaine abusers (Volkow et al., 1999). This phenomenon merits further investigation to determine hemispheric lateralization effects in addiction.

On the other hand, we found that increased FC between the VMN and auditory networks was associated with more severe anxiety symptoms in the MAs. This finding concurs with reports that the synchronization of functional brain connectivity in MAs is disrupted (Ahmadlou, Ahmadi, Rezazade, & Azad‐Marzabadi, 2013). Hyper‐synchronization in the brain suggests less efficient information communication across brain networks in patients with more severe affective symptoms. Therefore, our results indicate patients with severer anxiety symptoms may have experienced more critical detrimental effects on the brain functions.

Notably, we did not find association between affective symptoms and FC showing group differences in METH abusers. The possible reason is that the abstinence periods and severity of affective symptoms of the patients are varied and a part of the patients did not report any affective symptoms during the experimental session. Future studies with longitudinal investigations including short‐term and long‐term abstinence with affective symptom measures should be considered to further verify the neural substrates associated with METH induced affective symptoms.

### 4.3 Limitations

This study has several limitations. First, this study is a cross‐sectional investigation, and cannot establish causal relationship between brain function and drug addition, as well as its relationship with affective symptoms in the MAs. Thus, longitudinal studies are needed to help address these questions in future. Second, the subjects were recruited from a compulsory isolation and rehabilitation center and several unmeasured factors might have contributed to between‐group brain network differences, such as living environment, nutrition, and activity levels. Third, we only recruited male MAs and the results cannot represent the brain alterations in female patients. Since previous studies have demonstrated gender effects on brain disturbances in MAs, future works on female patients are needed. Fourth, subjects' education durations were not matched between the two groups, mainly due to difficulties in recruiting healthy young subjects with low educational levels in China currently. Although linear regression was used to exclude the potential effects produced by the education durations variances on the results a better designed community‐based study with a larger sample size should be conducted in the future to validate the functional neural mechanisms for affective symptoms associated with METH abuse. Finally, we could not identify and exclude subjects who fell asleep during eyes‐closed rs‐fMRI data acquisition, which may have influenced on our final results. Therefore, rs‐fMRI data obtained with subjects eyes open are needed to confirm our observation in the future.

## 5 CONCLUSION

By investigating whole‐brain network FC differences between MAs and HCs and characterizing affective symptoms severity associated with them in the MAs, we have identified changes in intra‐network FC of cerebellum and posterior cingulate cortex in the cerebellar and posterior salience networks respectively. Furthermore, we have demonstrated that the rostral ACC is a key region underlying the association between abstinence duration and affective symptom severity. Our findings shed light onto alterations in brain functional networks that underlie METH abuse and its withdrawal psychiatric symptoms and indicate that the rostral ACC may serve as a neural substrate in the association between abstinence length and affective symptom severity in MAs.

## CONFLICT OF INTERESTS

All authors declare no potential conflicts of interest.

## AUTHOR CONTRIBUTIONS

Ping Jiang, Jing Li, Xiaoqi Huang, and Qiyong Gong contributed to conception and study design. Jiayu Sun, Xiaobo Zhou, Ping Jiang, Lu Lu, Lei Li contributed to the data acquisition. Ping Jiang contributed to the statistical analysis and drafted the manuscript. Keith Kendrick contributed to the interpretation of the data and made critical revision of the paper. All authors contribute to interpret the results and gave final approval of the version to be published.

## Supporting information


**Appendix** S1: Supporting Information

## Data Availability

The data that support the findings of this study are available from the corresponding author upon reasonable request.
