# Supplementary material for: Functional connectivity abnormalities underlying mood disturbances in male abstinent methamphetamine abusers
Source: Hum Brain Mapp. 2021 May 3;42(11):3366–78. doi: 10.1002/hbm.25439 (PMC8249885; doi:10.1002/hbm.25439)
Supplement: Supplementary file 1 — Appendix S1: Supporting Information [file HBM-42-3366-s001.doc]

**Supplemental Material**

**Methods**

**Data denoising by FMRIB’s ICA-based Xnoiseifier**

To control for head motion and other nuisance noise (e.g., respiration, cardiac pulse), the FMRIB’s ICA-based Xnoiseifier—FIX (v1.061 beta) (Griffanti et al., 2014; Salimi-Khorshidi et al., 2014) was applied on the individual resting state fMRI data, which underwent four steps. First, all individual resting state fMRI data were analyzed with ICA using the MELODIC package in FSL (<https://fsl.fmrib.ox.ac.uk/fsl/fslwiki/MELODIC>) by probabilistic independent component analysis (PICA) (Beckmann & Smith, 2004). The number of independent components (ICs) was automatically estimated using the Laplace approximation as implemented in MELODIC (Beckmann & Smith, 2004). Second, to create a training dataset for FIX, one of the authors (P. J.) manually labeled the components into ‘signal’ and ‘noise’ from a sample of our datasets combining patients and controls based on both the spatial and temporal characteristics. Thereafter, the leave-one-out (LOO) approach was used to evaluate the accuracy of the hand-classified data. The results provided an overall high accuracy with mean true-positive rates at 98.1% and true-negative rates at 90.8%. Third, with the aid of the training dataset, FIX automatically classified single-session ICA output into ‘good’ and ‘bad’ components in the remaining data. Finally, the bad components and motion confounds with 24 motion parameters were regressed out from the preprocessed 4D fMRI data to obtain the cleaned datasets for the subsequent analyses.

**Pipeline for group comparison of brain network functional connectivity**

The following steps illustrate the pipeline of the fMRI data analysis for group comparisons of brain network functional connectivity (FC) between methamphetamine abusers (MAs) and healthy controls (HCs):

1. Preprocessing for individual resting state fMRI data includes brain extraction, excluding the first 5 volumes, motion correction, slice timing correction, high-pass temporal filtering equivalent to 100 s (0.01 Hz) and spatial smoothing with 5 mm FWHM Gaussian kernel.

2. FSL’s FIX described above was used to denoise the preprocessed individual fMRI data. The denoised fMRI data were used for the subsequent group-level FC analyses.

3. The group-level decomposition of resting state fMRI data for all the 48 HCs into brain networks with a determined number of components (i.e. 25 components) was performed using FSL’s MELODIC to produce the spatial templates for the resting state brain networks.

4. 19 out of 25 components were identified as signal networks from the HC group ICA (Figure S1), and used as spatial templates for dual-regression analyses to generate subject-specific versions of the spatial maps and associated time-series for each resting state brain network in each subject.

5. L1 regularised partial correlations of FC strengths between networks during resting state were generated by the FSLNets toolbox (http://fsl.fmrib.ox.ac.uk/fsl/fslwiki/FSLNets).

6. Group differences in resting state fMRI data were analyzed using FSL’s Randomise tool. The input for the intra- and between-network analyses were the subject-specific time-series from dual regression.

7. Individual mean z-scores of each network derived by dual regression representing intra-network FC strength and the transformed z-scores calculated by the L1 regularised partial correlation analyses in FSLNets representing between-network FC strength were used for correlation analysis with demographic and clinical measurements.

**Results**

**Figure S1: The resting state networks.**


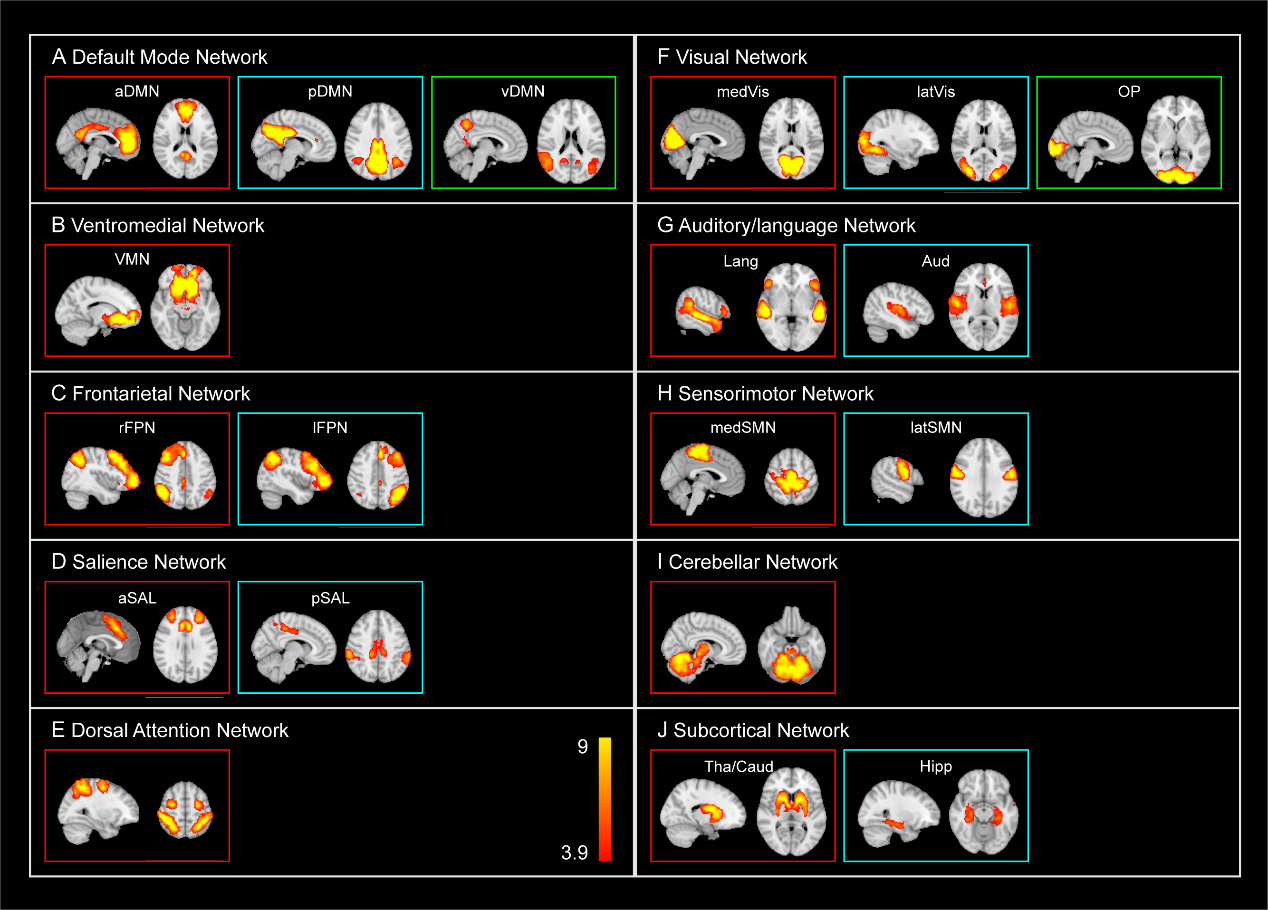


The spatial maps of brain networks (thresholded at Z > 3.9) shown here represent anterior, posterior and ventral subnetworks of the default mode network (a/p/v DMN), the ventromedial (VMN), frontoparietal (FPN), salience (SAL), dorsal attentional (DAN), visual (Vis), auditory (Aud), language (Lang), sensorimotor (SMN), cerebellar and subcortical networks. The spatial maps are displayed in sagittal and axial views and superimposed on the MNI152 standard space template image. R, right; l, left; a, anterior; p, posterior; med, medial; lat, lateral; OP, occipital pole; Tha, thalamus; Caud, caudate; Hipp, hippocampus.

**Figure S2: Intra-network FC of clusters within VMN showing significant correlations with PCA1 scores in the MAs**


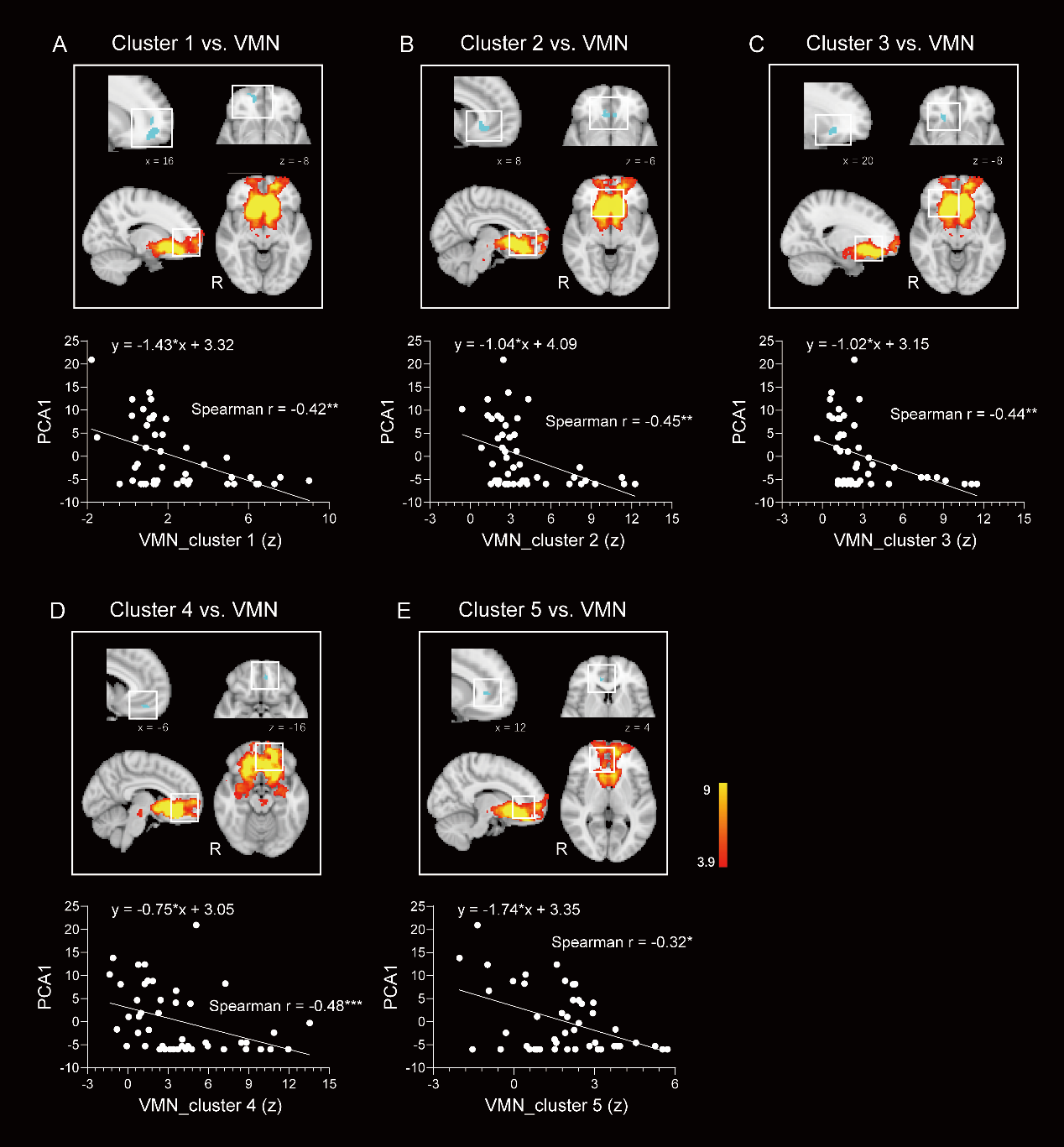


The figures and graphs demonstrate the intra-network FC of five clusters within the VMN showing significant correlations with PCA1 scores. VMN, ventromedial network; PCA1, the first component of principle component analysis; R, right.

**Figure S3: Significant correlation between affective symptoms and abstinence duration in the MAs**

**
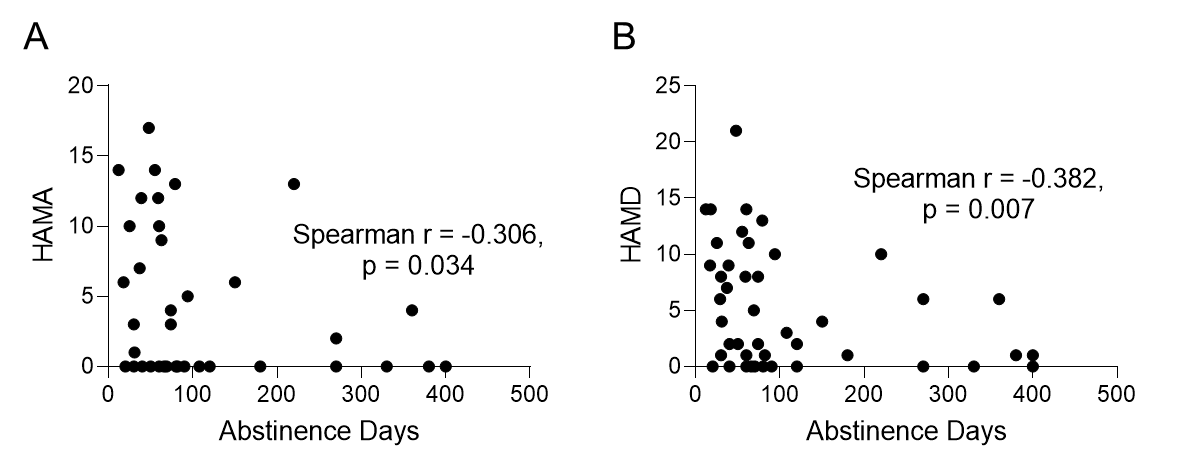
**

Results of Spearman correlation analyses between (A) anxiety and (B) depressive symptoms and number of abstinence days in the MAs. MA, methamphetamine abuser; HAMA, Hamilton Anxiety Rating Scale; HAMD, Hamilton Depression Rating Scale.

**Table S1.1: The mediation effect of within-network FC of rACC on the association between abstinence length and the level of anxiety symptoms in the MAs.**

| Variables | Model 1 (HAMA) | |  | Model 2 (FC of rACC within VMN) | |  | Model 3 (HAMA) | |
| --- | --- | --- | --- | --- | --- | --- | --- | --- |
|  | *β* | *t* |  | *β* | *t* |  | *β* | *t* |
| Age | -.098 | -0.66 |  | 0.075 | 0.52 |  | -0.063 | -0.47 |
| EduYear | 0.046 | 0.31 |  | 0.045 | 0.31 |  | 0.067 | 0.50 |
| AbsDay | -0.25 | -1.75^†^ |  | 0.34 | 2.40^*^ |  | -0.098 | -0.70 |
| rACC |  |  |  |  |  |  | -0.46 | -3.28^**^ |
| *R^2^* | 0.075 | |  | 0.12 | |  | 0.26 | |
| *F* | 1.20 | |  | 2.05 | |  | 3.78^*^ | |

N = 48. Each column is a regression model that predicts the criterion at the top of the column. AbsDay, abstinence days; EduYear, education years; FC, functional connectivity, HAMA, Hamilton Anxiety Rating Scale; MA, methamphetamine abuser; rACC, rostral anterior cingulate cortex; VMN, ventromedial network.

^†^ *p* < 0.10; ^*^ *p* < 0.05; ^**^ *p* < 0.01

**Table S1.2: Bootstrapping mediation effects**

| Effects | Coefficients | Boot_SE | Bootstrap 95% CI | | Coefficient ratio |
| --- | --- | --- | --- | --- | --- |
|  |  |  | Upper threshold | Lower threshold |  |
| Total effects | -0.254 | 0.10 | -0.455 | -0.050 |  |
| Direct effects | -0.0981 | 0.11 | -0.28 | 0.14 | 39.6 % |
| Indirect effects | -0.156 | 0.096 | -.038 | -0.0091 | 61.4 % |

Boot_SE: standard error for bootstrapping test; CI, confidence interval.

**Table S2.1: The mediation effect of within-network FC of rACC on the association between abstinence length and the level of PCA representing the affective symptoms in the MAs.**

| Variables | Model 1 (PCA1) | |  | Model 2 (FC of rACC within VMN) | |  | Model 3 (PCA1) | |
| --- | --- | --- | --- | --- | --- | --- | --- | --- |
|  | *β* | *t* |  | *β* | *t* |  | *β* | *t* |
| Age | -0.061 | -0.42 |  | 0.086 | 0.59 |  | -0.025 | -0.19 |
| EduYear | 0.11 | 0.75 |  | 0.053 | 0.36 |  | 0.13 | 0.98 |
| AbsDay | -0.31 | -2.17^*^ |  | 0.32 | 2.28^*^ |  | -0.17 | -1.26 |
| rACC |  |  |  |  |  |  | -0.42 | -3.01^**^ |
| *R^2^* | 0.11 | |  | 0.11 | |  | 0.26 | |
| *F* | 1.80 | |  | 1.90 | |  | 3.85^**^ | |

N = 48. Each column is a regression model that predicts the criterion at the top of the column. rACC, rostral anterior cingulate cortex; VMN, ventromedial network; AbsDay, abstinence days; EduYear, education years; FC, functional connectivity, HAMA, Hamilton Anxiety Rating Scale; MA, methamphetamine abuser; PCA1, the first component of principle component analysis.

^*^ *p* < 0.05; ^**^ *p* < 0.01

**Table S2.2: Bootstrapping mediation effects**

| Effects | Coefficients | Boot_SE | Bootstrap 95% CI | | Coefficient ratio |
| --- | --- | --- | --- | --- | --- |
|  |  |  | Upper threshold | Lower threshold |  |
| Total effects | -0.310 | 0.11 | -0.52 | -0.092 |  |
| Direct effects | -0.175 | 0.10 | -0.36 | 0.041 | 56.5 % |
| Indirect effects | -0.135 | 0.090 | -0.34 | -0.0001 | 43.5 % |

Boot_SE: standard error for bootstrapping test; CI, confidence interval.

Reference

Beckmann, C. F., & Smith, S. M. (2004). Probabilistic independent component analysis for functional magnetic resonance imaging. *IEEE Trans Med Imaging, 23*(2), 137-152. doi: 10.1109/TMI.2003.822821

Fagerstrom, K.O., Measuring Degree of Physical-Dependence to Tobacco Smoking with Reference to Individualization of Treatment. *Addictive Behaviors*, 1978. 3(3-4): p. 235-241.

Griffanti, L., Salimi-Khorshidi, G., Beckmann, C. F., Auerbach, E. J., Douaud, G., Sexton, C. E., . . . Smith, S. M. (2014). ICA-based artefact removal and accelerated fMRI acquisition for improved resting state network imaging. *Neuroimage, 95*, 232-247. doi: 10.1016/j.neuroimage.2014.03.034

Salimi-Khorshidi, G., Douaud, G., Beckmann, C. F., Glasser, M. F., Griffanti, L., & Smith, S. M. (2014). Automatic denoising of functional MRI data: combining independent component analysis and hierarchical fusion of classifiers. *Neuroimage, 90*, 449-468. doi: 10.1016/j.neuroimage.2013.11.046
